# Assessing the effect of mutant JAM-A overexpression on downstream signalling in breast cancer cells

**DOI:** 10.1186/1753-6561-9-S1-A47

**Published:** 2015-01-14

**Authors:** JSM Chu, E McSherry, K Brennan, A Hopkins

**Affiliations:** 1 Royal College of Surgeons in Ireland, 123 St. Stephen’s Green, Dublin 2, Ireland; 2 Department of Surgery, Royal College of Surgeons in Ireland, RCSI Education and Research Centre, Beaumont Hospital, Dublin 9, Ireland

## Background

Breast cancer causes the most number of deaths in females worldwide.[1] It contributed to 14% of cancer deaths in 2008 and is also the most commonly diagnosed cancer in women globally. Overexpression of the tight junction protein junctional adhesion molecule-A (JAM-A) in breast cancer tissue is known to produce less favourable outcomes in breast cancer patients. Overexpressed JAM-A may accomplish this by influencing downstream signalling of key proteins involved in cell proliferation, migration and the reaction of cells to therapeutic agents. This study aims to further investigate the promising role of JAM-A in breast cancer advancement by assessing the consequences of changing the structure of JAM-A on 10 key signalling proteins related to tumour progression.

## Methods

Three MDA-MB-231 cell lines were cultured. Two of the cell lines overexpressed JAM-A mutants, one with the first IgG loop deleted (DL1) and another with mutations at E61A and K63A (6163). The third was an empty vector cell line with normal levels of non-mutated JAM-A (EV). Preliminary Western blotting studies were used to screen cell lines for 10 key signalling proteins associated with tumour aggressiveness. The optical density for each band formed on film were analysed using ImageJ software. For each signalling protein, the density reading for each overexpressed cell line was compared against the EV cell line that was given a base value of ‘1’.

## Results

Overexpression of mutant JAM-A (DL1) resulted in increased levels of PAR6, p38, phospho-p38, aPKC, β1 integrin, pERK, pAKT and PAR3 signalling proteins. PTEN and ERK levels were essentially unchanged. Overexpression of mutant JAM-A (6163) resulted in increased levels of p38, phospho-p38 and pERK; while PAR6, aPKC, β1 integrin, ERK, pAKT and PAR3 all showed decreased levels.

## Conclusions

Our data in breast cells show that mutation of JAM-A can alter the levels of 10 key signalling proteins. In various studies conducted, increased levels of Par6, p38, phospho-P38, aPKC, beta1 integrin, ERK, pERK and pAkt may facilitate tumourigenicity, while decreased levels of PAR3 and PTEN in cells may do likewise. Thus the changes in signalling protein levels acquired from our data suggest that mutant JAM-A (DL1) assists the development of malignancy in breast cancer cells, however mutant JAM-A (6163) may not have the same effect. There is currently a scarce pool of knowledge on the role of JAM-A in breast cancer advancement and our study provides a rationale for further investigation into this relationship.

